# Impact of Alkyl Chain Length on the Formation of Regular- and Reverse-Graded Quasi-2D Perovskite Thin Films

**DOI:** 10.1021/acsmaterialslett.3c01073

**Published:** 2023-12-19

**Authors:** Alessandro Caiazzo, Kunal Datta, Laura Bellini, Martijn M. Wienk, René A. J. Janssen

**Affiliations:** † Molecular Materials and Nanosystems and Institute of Complex Molecular Systems, Eindhoven University of Technology, P.O. Box 513, 5600 MB Eindhoven, The Netherlands; ‡ Dutch Institute for Fundamental Energy Research, De Zaale 20, 5612 AJ Eindhoven, The Netherlands

## Abstract

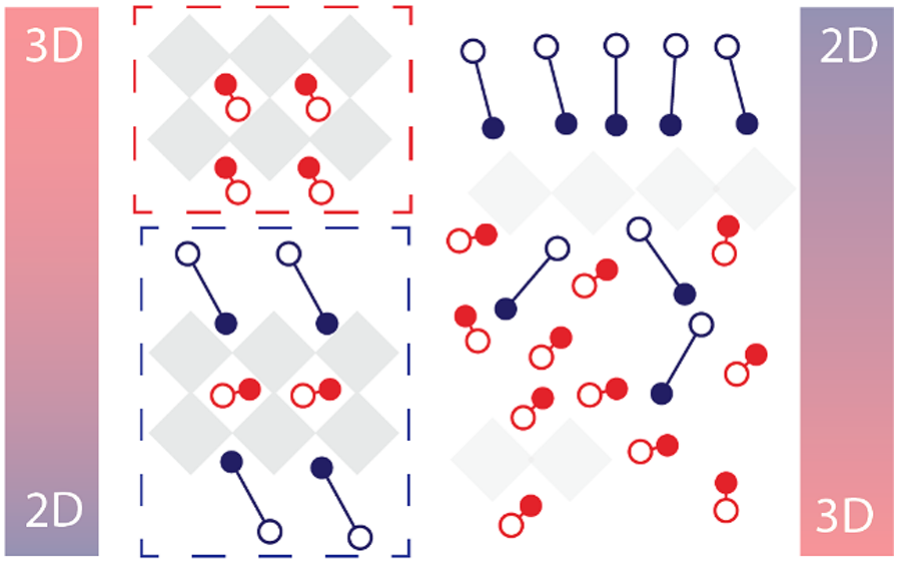

Crystallization of low-dimensional perovskites is a complex process that leads to multidimensional films comprising two-dimensional (2D), quasi-2D, and three-dimensional (3D) phases. Most quasi-2D perovskite films possess a regular gradient with 2D phases located at the bottom of the film and 3D phases at the top. Recently, multiple studies have reported reverse-graded perovskite films, where the location of the 2D and 3D structures is inverted. The underlying reasons for such a peculiar phase distribution are unclear. While crystallization of regular-graded quasi-2D perovskites has been described as starting with 3D phases from the liquid–air interface, the film formation of reverse-graded films has not been investigated yet. Here, we examine the impact of the alkyl chain length on the formation of regular- and reverse-graded perovskites using *n-*alkylammonium ions. We find that long alkyl chains reverse the phase distribution gradient. By combining photoluminescence spectroscopy with *in situ* optical absorption measurements, we demonstrate that crystallization starts at the liquid–N_2_ interface, though as 3D phases for short-chain *n-*alkylammonium ions and as quasi-2D phases for long chains. We link this behavior to enhanced van der Waals interactions between long-chain *n-*alkylammonium ions in polar solvents and their tendency to accumulate at the liquid–N_2_ interface, creating a concentration gradient along the film thickness.

Quasi-two-dimensional (quasi-2D) metal-halide perovskites are promising candidates for a variety of optoelectronic devices, such as solar cells, light-emitting diodes, and photodetectors.^1−3^ Conventional three-dimensional (3D) metal-halide perovskites possess an ABX_3_ structure, where A is a small monovalent cation, such as methylammonium (MA) or formamidinium (FA), B is usually a divalent lead (Pb) or tin (Sn) ion, and X is a halide anion, usually iodide (I) or bromide (Br).^4^ These structures consist of metal-halide octahedra intercalated by small cations. Ruddlesden–Popper 2D and quasi-2D perovskites share a similar inorganic part but with a large ammonium spacer cation, such as butylammonium (BA) or phenethylammonium (PEA), cleaving the octahedra along the ⟨100⟩ direction to form a layered crystal structure.^5^ 2D perovskites (R_2_BX_4_, where R is an ammonium spacer cation) possess a single layer of BX_6_^4–^ octahedra sandwiched between ammonium spacer cations (*n* = 1), whereas quasi-2D perovskites (R_2_A_*n*-1_B_*n*_X_3*n*+1_) have more layers of BX_6_^4–^ octahedra in between (*n* = 2–5).^1,6^

The crystallization of quasi-2D perovskites is more complex than for pure 3D or 2D.^7−9^ For instance, for BA_2_MA_3_Pb_4_I_13_ (*n* = 4), the precursor solution contains BAI, MAI, and PbI_2_, which compete to crystallize 2D, quasi-2D with different *n* values, and 3D perovskites.^10^ The overall result is a mixed-phase quasi-2D perovskite film with an average ⟨*n*⟩ value. Quasi-2D perovskites largely show a phase distribution gradient with (quasi-)2D phases at the bottom of the film and 3D phases at the top (regular-graded).^11−20^ Phase distribution can be tuned via solvent and additive engineering,^12,21−23^ modifying the substrate surface, or by varying processing conditions.^24−26^ Of course, the ammonium spacer cation also has a profound effect on which phases are formed during crystallization. Its chemical structure influences interspacer interactions and, as a result, the phase distribution of the quasi-2D perovskite film.^27^ For example, fluorination of PEA reduces the formation of *n* = 1 phases, which is usually detrimental for photovoltaic performances, and allows a more efficient charge transport because of a different molecular stacking.^15,28^ Recently, so-called reverse-graded quasi-2D perovskites have been reported, where 3D phases are at the bottom (substrate interface) and (quasi-)2D phases are at the top (air or N_2_ interface) of the film. The first studies to show this peculiar behavior employed cyclohexylmethylammonium iodide (CMAI) as a spacer.^29,30^ Both Type I (i.e., nested) and Type II (i.e., cascading) alignments of the valence and conduction bands have been reported for graded quasi-2D films.^17,30−33^ A Type II phase distribution of regular-graded quasi-2D films is suitable for solar cells in a *p-i-n* configuration because it facilitates efficient hole and electron collection, leading to power conversion efficiencies (PCEs) up to ∼21%.^34−36^ Conversely, for reverse-graded quasi-2D perovskites, the Type II band alignment is better suited for *n-i-p* devices, and the highest reported PCEs are ∼20%.^30,32,37^

Although regular- and reverse-graded quasi-2D perovskites have been reported before, their crystallization mechanism and the origin of this distribution are still unclear. Mao et al. claimed that BA ions prefer to remain in solution, while MA ions stay at the surface, thus explaining the 2D-3D gradient that is often observed.^38^ Liu et al. employed a cation diffusion model to explain phase distribution in quasi-2D perovskite films and found large differences in diffusivity between cations based on their mass and molecular volume.^39^ They mostly attributed differences in cation diffusivity as the main reason behind the formation of a 2D-3D graded film. Jang et al. studied the formation mechanism of multiphase Ruddlesden–Popper perovskites for isobutylammonium (isoBA) using cold- and ambient-antisolvent bathing after spin coating, prior to annealing.^37^ They found that cold-bathing results in small-*n* phase formation at the top of the film and considered that at low temperature delayed sequential nucleation occurs, where the lower solubility of the small (MA) compared to the bulky (isoBA) organic cations favors the formation of 3D phases as the substrate–liquid interface.

In this work, we analyze a series of quasi-2D perovskite films for *n*-alkylammonium ions with increasing chain length, going from *n*-butylammonium (C4) to *n*-dodecylammonium (C12). We observe that from *n*-octylammonium (C8) onward a reversal of the phase distribution gradient is obtained, hinting that the length and apolar nature of the alkyl chains have a profound impact on the crystallization of the film. Correlating photoluminescence (PL) spectra with *in situ* UV–vis–NIR absorption spectroscopy during thermal annealing to reveal the temporal evolution of the crystallization of quasi-2D perovskites,^12^ we find that crystallization begins at the liquid–N_2_ interface for both regular- and reverse-graded perovskites; however, the crystallization starts as quasi-3D for short *n*-alkylammonium ions, and as quasi-2D perovskites for long *n*-alkylammonium ions. We finally use a mixture of short and long *n*-alkylammonium ions to tune the stratification of 2D and 3D phases and find that it also favorably influences the film morphology.

We fabricated quasi-2D perovskite films with the formula (R)_2_MA_3_Pb_4_I_13_, where R = C4, C5, C6, C8, or C12 ammonium iodide. Based on our previous work, we optimized the solvent/cosolvent (DMF/DMSO) ratio to 4:1 such that large amounts of small-*n* phases (*n* = 1, 2, 3, etc.) are formed.^12^ While the presence of these phases has been shown to be detrimental to photovoltaic performance because of unfavorable parallel orientation and consequently poor charge transport,^40^ we opted for this phase distribution to more easily observe differences between 3D and 2D or quasi-2D phases and their distribution. Figure 1a displays the first derivative of the UV–vis–NIR absorption spectra of such films (the full spectra are shown in Figure S1, Supporting Information). Structural phases such as *n* = 1–5 are usually observed as shoulders in the absorption spectra;^12^ thus, the first derivative will highlight such peaks. All films display a negligible peak at >750 nm, which indicates a very shallow onset for the absorption of 3D perovskite phases. With increasing length of the *n*-alkylammonium ion, the phase distribution shifts toward smaller-*n* phases as can be seen from the blue shift of the spectral signatures. For instance, C4 shows predominantly the *n* = 3 (∼600 nm) phase and small amounts of *n* = 2 (∼570 nm) and *n* = 4 (∼640 nm) phases, while the amount of *n* = 2 is enhanced from C5 onward. C8 forms mostly *n* = 2, whereas C12 is the only *n*-alkylammonium ion that also forms *n* = 1 (∼520 nm). The formation of smaller-*n* phases with longer alkyl chains can be attributed to their enhanced van der Waals interactions, which facilitate the formation of small-*n* phases because the assembly of alkyl chains becomes more energetically favored. This scenario is comparable to the use of aromatic spacers; these molecules, such as PEA, can form π–π stacking, and such an additional interaction leads to smaller-*n* phases being more easily formed (Figure S2, Supporting Information).

**Figure 1:**
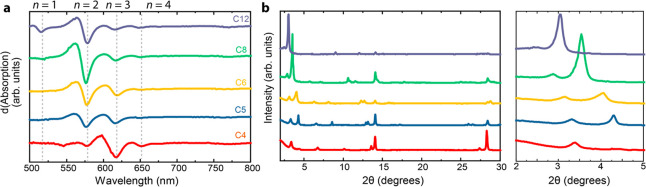
(a) First derivatives of UV–vis–NIR absorption spectra for R_2_MA_3_Pb_4_I_13_ films where R = C4–C12. (b) XRD patterns for the same films as in (b) with the inset focused on 2–5°.

X-ray diffraction (XRD) confirms the trend observed with UV–vis–NIR absorption spectroscopy (Figure 1b). Parallel-oriented 2D and quasi-2D phases display diffraction peaks at 2θ angles below 10°. C4 shows the formation of *n* = 3 (3.4°) and *n* = 2 (4.4°). These two diffraction peaks are visible for all the films; but *n* = 3 steadily decreases, while *n* = 2 becomes dominant in the XRD pattern with an increasing alkyl spacer length.^12^ The diffraction angles for both quasi-2D phases decrease from C4 to C12, in line with an increase in lattice spacing because of the presence of a larger organic cation. The 3D perovskite peaks at 14° and 28° are observed for all films. C4 shows the strongest diffraction at the above-mentioned angles, and C12 shows the weakest, consistent with the observation that longer alkylammonium ions form more 2D and quasi-2D phases.

To investigate the impact of alkyl chain lengths on the phase distribution gradient, we analyzed the locations of 2D and 3D phases via PL spectroscopy (Figure 2). C4 displays relatively small differences between top (exciting at the film–N_2_ interface) and bottom (exciting at the film–substrate interface) excitations, which reveal a rather uniform layer with little vertical distribution of different *n*-phases. In contrast, C5 and C6 exhibit the characteristic features of a 2D-3D, regular-graded perovskite, where quasi-2D phases are at the bottom of the film, and 3D phases are at the top. A variety of *n*-phases can be identified, with peaks at 575 (*n* = 2), 615 (*n* = 3), and 650 nm (*n* = 4), when exciting the film from the bottom side. On the contrary, when exciting from the top side, mainly 3D perovskite phases emit at ∼750 nm. Remarkably, the opposite behavior is observed for films prepared by using long-chain cations. Starting from C8, the overall phase distribution becomes 3D-2D, i.e., reverse-graded. In this case, *n* = 2 phases (570 nm) emit mostly from the top side of the film, whereas 3D phases are prevalent at the bottom. This behavior is even more apparent for C12 where the PL of the *n* = 2 phase at 570 nm remains higher than that from the 3D phase at 765 nm under top illumination. These results demonstrate that the gradient of the 2D and 3D phases can be tuned by increasing the length of the alkyl spacer (Figure 2c). This is possibly linked to the surfactant properties of long *n*-alkylammonium ions, that preferentially locate at the liquid-N_2_ interface.^41^ Also the formation energy will vary with the nature of R for the R_2_MA_3_Pb_4_I_13_ perovskites. In addition, the reduced solubility of long *n*-alkylammonium ions in the polar DMF/DMSO solvent mixture can enhance nucleation of small-*n* phases compared to the 3D phase.^37^ These mechanisms can explain why, for C8 and C12, the 2D phases crystallize mostly at the liquid-N_2_ interface. The solubility of the alkylammonium iodides in DMF decreases on going from C4 (11.1 M) to C12 (3.2 M). From the solubility trend, shown in Figure S3 (Supporting Information), we expect spacer salts with solubility lower than the one of C8 (∼8.1 M) to produce a reverse-graded film. The CMAI spacer shows a solubility of ∼6.7 M and falls below this limit, thus–as expected–it forms a reverse-graded film, as shown previously in the literature.^29^ We did not study the solubility of other spacer salts, but this solubility trend seems to explain the empirical observations in both this work and others based on CMAI.

**Figure 2:**
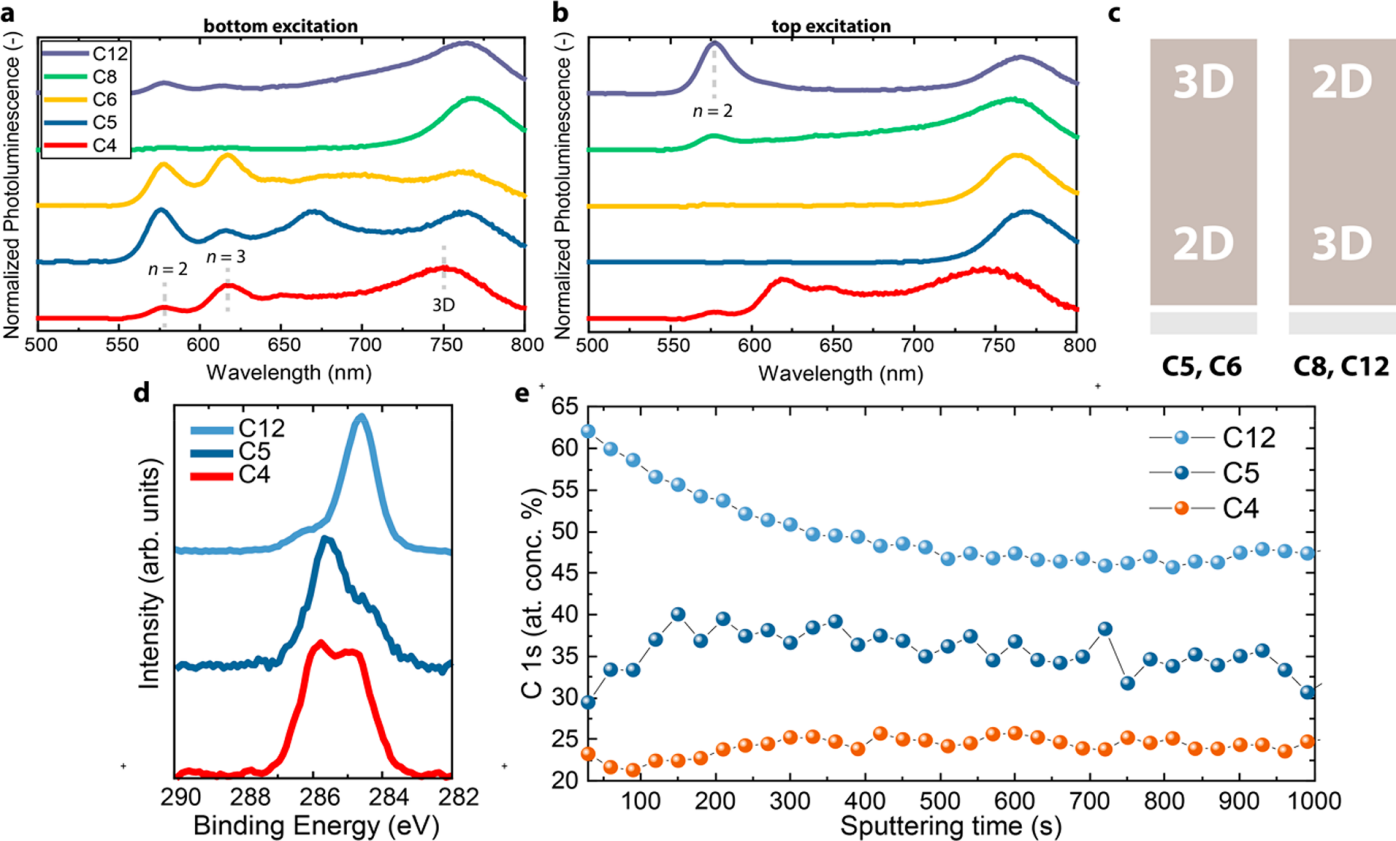
(a-b) Photoluminescence spectra of R_2_MA_3_Pb_4_I_13_ films, where R = C4–C12, recorded with bottom (a) and top (b) excitation at 405 nm. (c) Illustration of regular (left) and reversed (right) phase distribution gradients. (d) C 1s XPS spectra for (C4)_2_MA_3_Pb_4_I_13_, (C5)_2_MA_3_Pb_4_I_13_, and (C12)_2_MA_3_Pb_4_I_13_ films. (e) Atomic concentration of carbon for the same films as (a) as a function of sputtering time. After 1000 s, the entire film is etched.

We performed X-ray photoelectron spectroscopy (XPS) on C4, C5, and C12 films to further analyze the gradient distribution of the 2D and 3D phases. By analyzing the C 1s spectra (Figure 2d) from the film surface, we observe that C4 displays two features attributed to carbon atoms binding to either carbon (285 eV) or nitrogen (286 eV). For C5 and C12, the same features are observed, but the 285 eV signal is, expectedly, more intense in the case of C12, whereas the 286 eV peak is more intense for C5. We performed XPS depth-profiling using argon ion sputtering. Figure 2e displays the atomic concentration of C as a function of sputtering time. In the case of C12-based film, the C concentration is highest at the top of the film and then decreases with sputtering time and stabilizes at about 45%, in agreement with a reverse-graded film. In the case of C5, the carbon amount is lowest at the top of the film, as expected for a regular-graded film, and the reaches a maximum after which it remains fairly constant going deeper into the film. The C4 film shows a small initial increase, followed by a stable carbon content along the remainder of the film thickness.

Crystallization of quasi-2D perovskites has been shown to start from the liquid–air interface as a 3D perovskite phase for short *n*-alkylammonium cations and to form 2D or quasi-2D phases toward the bottom at a later stage,^10,12,42,43^ producing a 2D-3D gradient. Because long *n*-alkylammonium ions form 3D-2D reverse-graded Ruddlesden–Popper films, we investigated the crystallization mechanism using *in situ* UV–vis–NIR absorption during thermal annealing of wet films to reveal the origin of the differences between short and long *n*-alkylammonium ions. As displayed in Figure 3a, a film using C4 starts to crystallize as quasi-3D (unidentified large-*n* value) perovskites with an onset at ∼750 nm appearing after about 5 s. After 10 s, an excitonic peak related to *n* = 3 appears, indicating that quasi-2D perovskite phases are formed after the 3D phase has begun crystallizing. The film using C5 follows the same mechanism (Figure S4, Supporting Information). After 1 s, C6-based film starts crystallizing as *n* = 4 and *n* = 5 phases with peaks at ∼640 and ∼660 nm (Figure 3b); shortly afterward (∼5 s), it develops into a quasi-3D perovskite, with an onset at ∼770 nm, and finally, it develops *n* = 2 and 3 features. In contrast, C8- and C12-based films start crystallizing after 5 s mainly as *n* = 2 (∼570 nm), developing 3D-like features only long (∼30 s) after annealing has started (Figure 3c,d). Interestingly, this phenomenon is also visible to the naked eye. During annealing, C8 and C12 films turn from yellow (wet film) to red (wide bandgap small-*n* phase) and then to a darker color which indicates the formation of lower bandgap phases (3D) (Figure S5, Supporting Information). By comparison, the films turn immediately darker in the case of C4 (Figure S6, Supporting Information).

**Figure 3:**
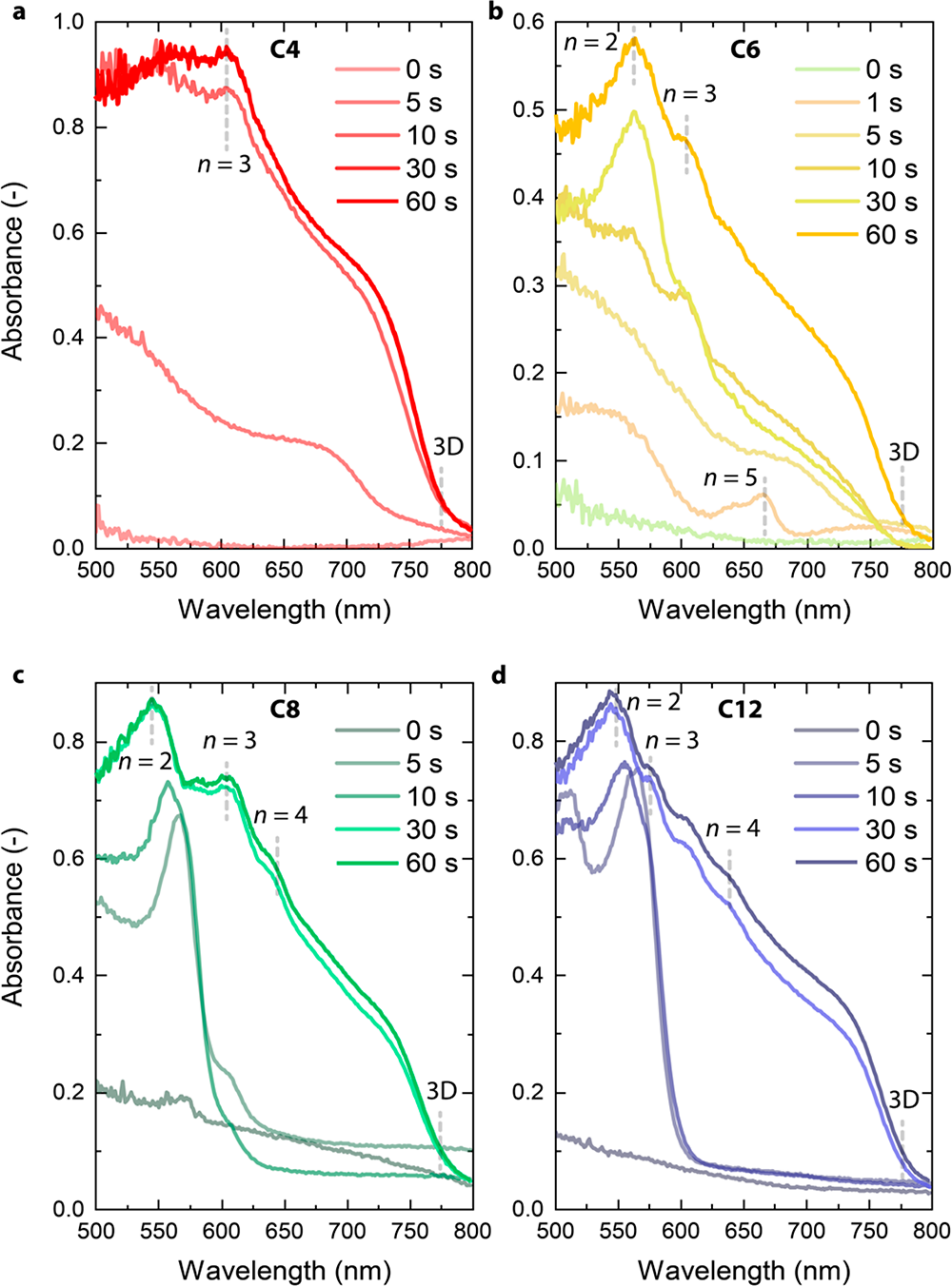
*In situ* UV–vis–NIR absorption spectra during thermal annealing of (R)_2_MA_3_Pb_4_I_13_. (a) R = C4. (b) R = C6. (c) R = C8. (d) R = C12.

We therefore find a striking trend between the *n*-alkylammonium ion structure and crystallization dynamics of the different phases. By increasing the alkyl length (C4 to C12), the crystallization shifts from starting as a higher-dimensional perovskite to starting as a quasi-2D. With the location of 3D phases being the top of the film, as demonstrated by PL spectroscopy, C4 and C6 confirm that the perovskite films crystallize as quasi-3D (or large-*n*) at the liquid–air interface. In the case of C6, we also expect *n* = 4 and *n* = 5 phases at the surface of the film, which might not be visible in PL because of efficient carrier transfer from high to low bandgap phases. For C8- and C12-based films, crystallization starts as quasi-2D (*n* = 2), again at the liquid–N_2_ interface. Irrespective of the perovskite phases formed, crystallization begins at the liquid–N_2_ interface. The experiments show that with long alkyl chains (C8–C12), crystallization of the 3D phase is significantly delayed (∼30 s) compared to films with short alkyl chain cations (∼5 s).

Interestingly, the cation diffusivity model proposed by Liu et al. does not agree with our experimental observations.^39^ In fact, the diffusivity of cations with larger mass, such as C5–C12, should decrease even further compared to C4 and MA, and the 2D-3D phase distribution gradient should be more evident. The diffusivity model also does not predict a reversed-graded phase distribution. Likewise, the model of Jang et al. cannot explain the results obtained here because crystallization of the 3D phase is retarded compared to the low-*n* quasi-2D perovskite in the reverse-graded perovskite films obtained with C8 and C12.^37^

Based on our experimental results, we postulate the following crystallization mechanism (Figure 4). We consider that crystallization starts at the liquid–N_2_ interface where the solids concentration increases as a result of solvent evaporation. With regard to C4, this spacer is similar to MA. The MA concentration at the liquid–N_2_ interface is high enough to drive the crystallization of 3D perovskites, shortly followed by quasi-2D phases. In this case, the phase distribution gradient is minimal. C5 falls in a similar category as C4, meaning that the crystallization will start as 3D. However, with increasing length, the *n*-alkylammonium ions could assemble more at the polar liquid–N_2_ interface, and the increased van der Waals interaction between the longer alkyl chains favors formation of quasi-2D crystalline nuclei. Such a self-assembly could be dictated by increasing the surfactant properties of *n*-alkylammonium ions with increasing alkyl-chain length. The C6 molecules are concentrated enough at the liquid–N_2_ interface to enable a crystallization of larger-*n* quasi-2D phases, namely *n* = 4 and 5. For C8 and C12 cations, this tendency is further enhanced such that crystallization at the liquid–N_2_ interface directly starts as a quasi-2D perovskite. As a result, the concentration of MA increases toward the bottom of the film, forming 3D perovskites at the interface with the substrate.

**Figure 4:**
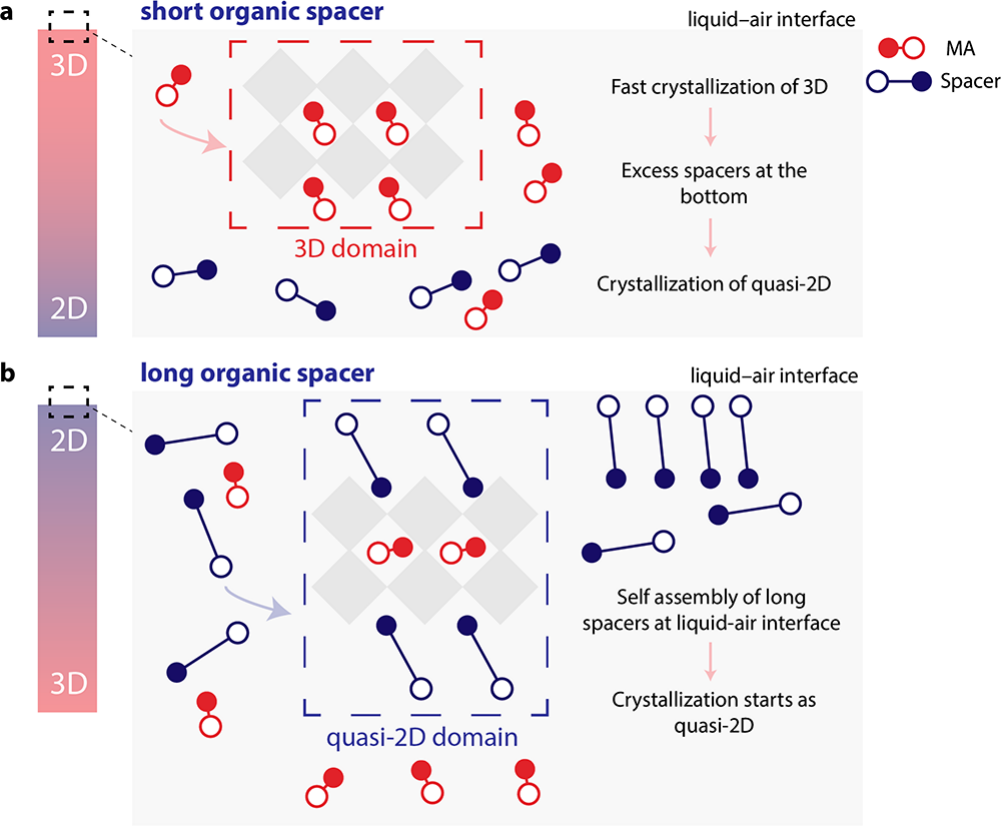
Illustration of the proposed crystallization mechanism for (a) short (C4, C5) and long (C6, C8, and C12) *n*-alkylammonium ions.

We confirmed the above-mentioned crystallization mechanism by mixing C4 and C12 in a 9:1 ratio to form a (C4_0.9_C12_0.1_)_2_MA_3_Pb_4_I_13_ Ruddlesden–Popper perovskite film. As mentioned, C12 molecules will locate at the liquid–N_2_ interface, and the crystallization of a mixed *n*-alkylammonium ion film is expected to be intermediate between one of the individual C4 and C12 components. *In situ* UV–vis–NIR during thermal annealing (Figure 5a) shows that the small amount of C12 present in the solution (10% compared to C4) is enough to lead the crystallization to start as a C12-based *n* = 2 (∼570 nm) in the first few seconds of annealing. After 5 s, the perovskite film already shows quasi-3D and quasi-2D phases, and such features develop further during annealing. Changes in the crystallization rate are usually effective in changing the morphology of the resulting film. Scanning-electron microscope (SEM) images show that the pristine C4 Ruddlesden–Popper perovskite film is not uniform but contains pinholes (Figure 5b). The mixed C4–12 films, however, display a uniform and pinhole-free film (Figure 5c).

**Figure 5:**
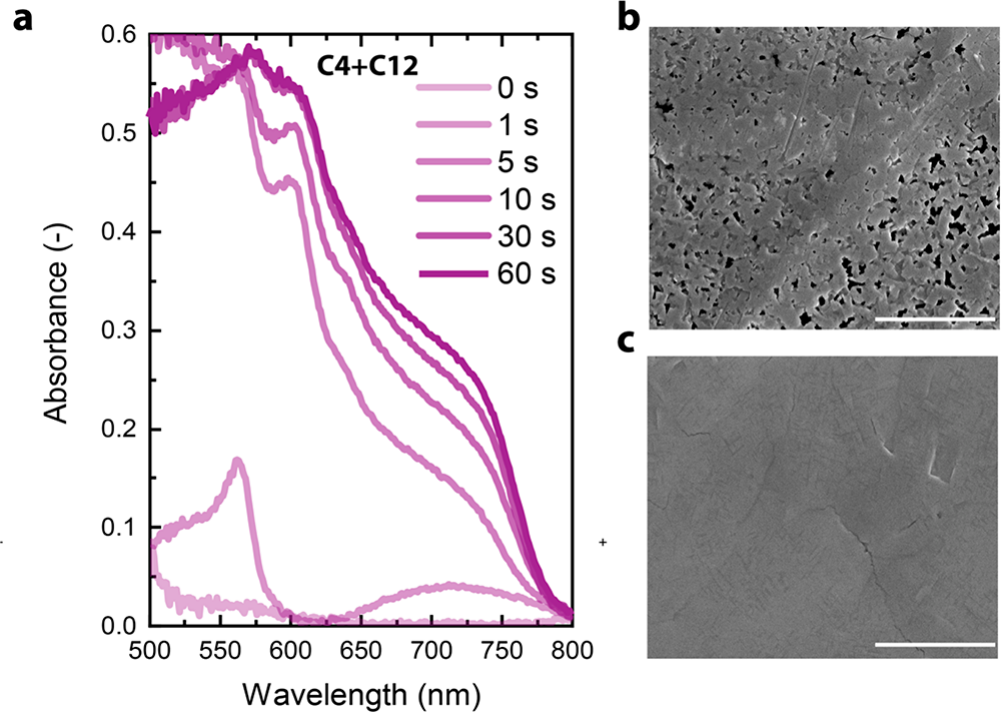
(a) *In situ* UV–vis–NIR absorption of (C4_0.9_C12_0.1_)_2_MA_3_Pb_4_I_13_ films during thermal annealing. (b–c) Top-view SEM images of (C4)_2_MA_3_Pb_4_I_13_ (b) and (C4_0.9_C12_0.1_)_2_MA_3_Pb_4_I_13_ (c). Scale bar is 4 μm.

Despite the above-mentioned multispacer system (C4 + C12) not leading to a phase-pure film, the use of mixed *n*-alkylammonium ions could prove itself useful to control the crystallization rate and the phase distribution gradient. Theoretically, an optimized multispacer system could pave the way for a phase-pure system, which has been a difficult task to achieve in quasi-2D perovskites. Recently, Guo et al. demonstrated the formation of a phase-pure quasi-2D perovskite with *n* = 3 and PEA as the spacer, by using a branched cospacer (triphenylmethylammonium) that inhibits the formation of small-*n* phases.^44^ Changes in the spacers’ molecular structure could also induce different phase distribution gradients. Multispacer systems have been used before in quasi-2D perovskites optoelectronic devices, although mostly by trial-and-error.^45^ With more understanding of the crystallization mechanism of quasi-2D perovskites based on multiple spacers, a new degree of tunability of Ruddlesden–Popper perovskites can be added, possibly leading to a new class of perovskite films containing spacers with a variety of chemical and optoelectronic properties.

We demonstrate that the length of *n-*alkylammonium ions in quasi-2D perovskites determines the phase distribution gradient, with short alkyl chains favoring regular-graded films (i.e., 3D at top and quasi-2D at bottom) and long alkyl chains resulting in reverse-graded films (i.e., quasi-2D at top and 3D at bottom). *In situ* UV–vis–NIR spectroscopy during thermal annealing showed that crystallization starts with the 3D phase for short alkyl chains but with the quasi-2D phases and for long alkyl chains. This implies that in each case crystallization starts at the liquid–N_2_ interfaces where the solid concentration increases as the solvent evaporates. We attribute the different behavior of short and long *n-*alkylammonium ions to the tendency of long apolar alkyl chains to accumulate at the liquid–N_2_ interface and the increasing van der Waals interactions between chains when their length increases.

## Experimental Section

### Starting Materials

PbI_2_ was purchased from TCI Chemicals (99.99%), and alkylammonium iodides were purchased from Great Cell Solar. Solvents were purchased from Sigma-Aldrich. All materials were used as received. To prepare R_2_MA_3_Pb_4_I_13_ solutions (⟨*n*⟩ = 4), a PbI_2_-based 1 M solution was prepared by mixing alkylammonium iodide (RAI), methylammonium iodide (MAI), and PbI_2_ in the ratio 2:3:4 in a *N,N*-dimethylformamide/dimethyl sulfoxide (DMF/DMSO) 4:1 (v/v) solvent mixture. RAI corresponds to *n*-butylammonium (C4), *n*-pentylammonium (C5), *n*-hexylammonium (C6), *n*-octylammonium (C8), and *n*-dodecylammonium (C12) iodide.

### Film Deposition

A 60 μL portion of the precursor solution was dropped onto a glass substrate (previously cleaned by sonication in isopropanol and by UV-ozone treatment for 30 min). Spin coating was performed at 5000 rpm for 45 s, followed by thermal annealing at 100 °C for 10 min. All fabrication was performed in an N_2_-filled glovebox.

### Film Characterization

UV–vis–NIR spectra were recorded by using a PerkinElmer Lambda 1050 UV–vis–NIR spectrophotometer. Photoluminescence spectra were recorded using an Edinburgh Instruments FLSP920 double-monochromator luminescence spectrophotometer. X-ray diffractograms were recorded by using a Bruker 2D phaser (Cu Kα radiation, λ = 1.5406 Å): measurements were performed in the range 3–40° with a step size 0.02° and collection time of 0.5 s. XPS measurements were performed using a Thermo Scientific K-Alpha with a 180° double focusing hemispherical analyzer and a 128-channel detector. Monochromatic Al Kα (1486.6 eV) radiation was used, and the X-ray spot size was 400 μm. For the surface analysis, a survey spectrum was first measured for 12 scans with a pass energy of 200 eV. High-resolution scans (20 times) of each element were conducted with a pass energy of 50 eV. During the sputtering experiment, the sample was removed layer-by-layer by argon ion etching operated at a low current and low ion energy (500 eV). The crater region generated by argon ions is ∼2 × 4 mm^2^. For the depth profiles, snapshot mode was used for each element, and the number of frames was 5 × 1 s. SEM images were collected with an FEI Quanta 3D FEG microscope (5 keV electron beam, secondary electron detector). Films were sputtered with Au beforehand to improve the surface conductivity.

### *In Situ* UV–Vis–NIR Absorption

White paint was applied on the back of a glass substrate; then, the substrate was placed on the hot plate in an N_2_-filled glovebox and illuminated by focused light from a halogen lamp. A fiber optical cable was placed at an off-specular angle and collected the light that was scattered by the white paint and transmitted through the perovskite layer. The fiber was connected to a spectrophotometer that analyzed the raw photon counts. The absorbance was calculated according to the following equation
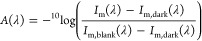
where *I*_m_(λ) represents the photon counts at wavelength λ, and *I*_m,dark_(λ) and *I*_m,blank_(λ) represent a dark and blank reference, respectively, on a similar substrate on the hot plate.

## Supporting Information Available

Additional UV–vis–NIR absorption and photoluminescence spectra, solubility data, and photographs of films during crystallization (PDF)
