# Supplementary material for: Impact of Alkyl Chain Length on the Formation of Regular- and Reverse-Graded Quasi-2D Perovskite Thin Films
Source: ACS Mater Lett. 2023 Dec 19;6(1):267–74. doi: 10.1021/acsmaterialslett.3c01073 (PMC10762656; doi:10.1021/acsmaterialslett.3c01073)
Supplement: Supplementary file 1 — tz3c01073_si_001.pdf [file tz3c01073_si_001.pdf]

Supporting Information

Impact of Alkyl Chain Length on the Formation of Regular- and Reverse-Graded Quasi-2D Perovskite Thin Films

Alessandro Caiazzo,[†] Kunal Datta,[†] Laura Bellini,[†] Martijn M. Wienk,[†] René A. J. Janssen*[†‡]

[†] Molecular Materials and Nanosystems and Institute of Complex Molecular Systems, Eindhoven University of Technology, P.O. Box 513, 5600 MB Eindhoven, The Netherlands
[‡] Dutch Institute for Fundamental Energy Research, De Zaale 20, 5612 AJ Eindhoven, The Netherlands
* Email: r.a.j.janssen@tue.nl

Additional Figures

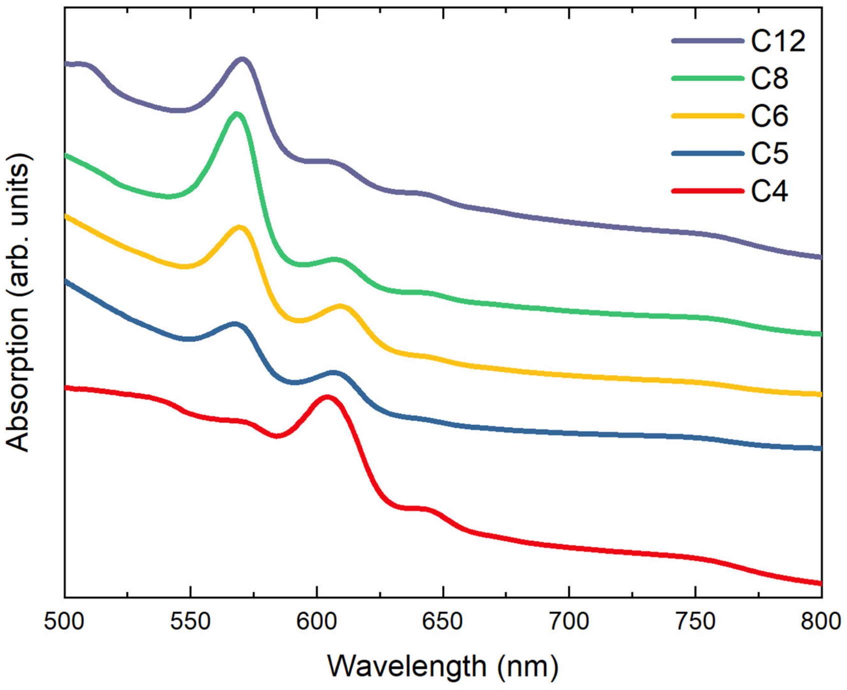

Figure S1. UV-vis-NIR absorption spectra for $R_2MA_3Pb_4I_{13}$ films, where R = C4–C12.

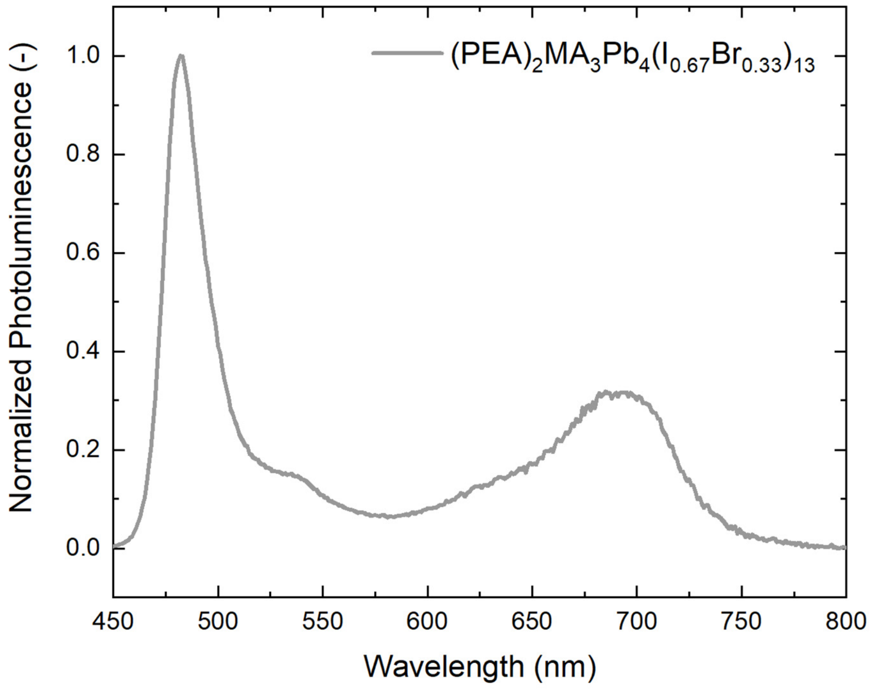

Figure S2. Photoluminescence spectrum of $(PEA)_2MA_3Pb_4(I_{0.67}Br_{0.33})_{13}$ recorded with bottom (glass) side excitation. With PEA as spacer, smaller-*n* phases such as $n = 1$ and $n = 2$ are more easily formed because of its intermolecular π–π interaction.

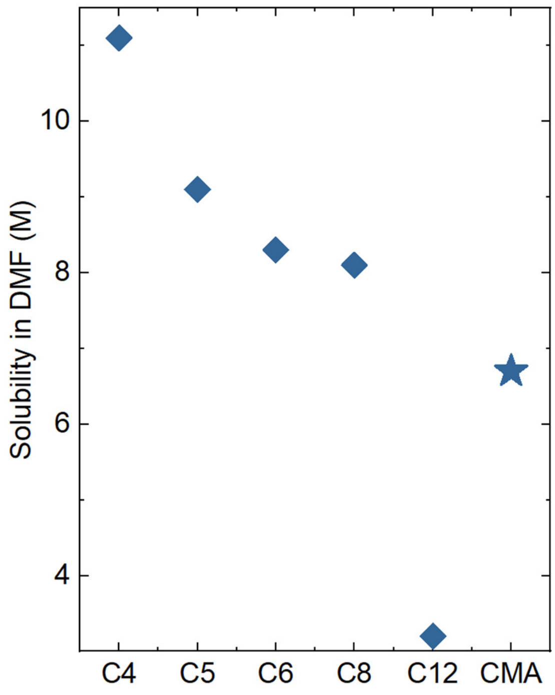

Figure S3. Solubility of C4, C5, C6, C8, C12 and CMA in DMF.

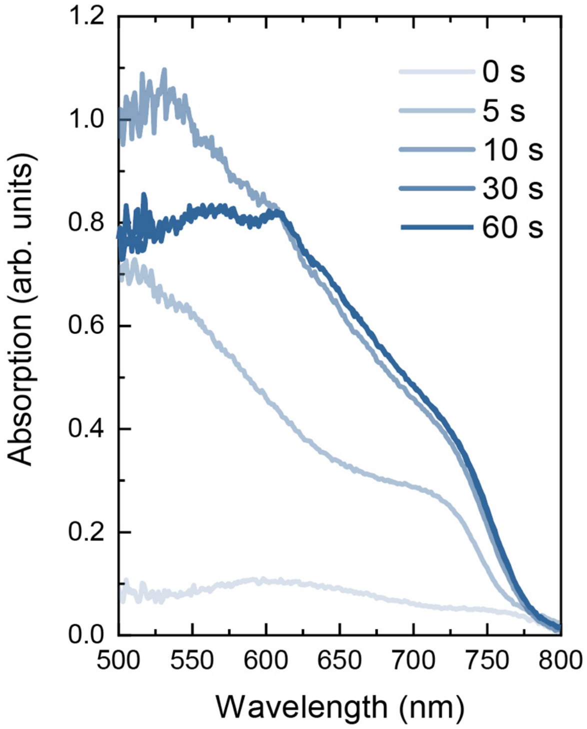

Figure S4. In-situ UV-vis absorption spectra during thermal annealing of $(R)_2MA_3Pb_4I_{13}$ where R = C5.

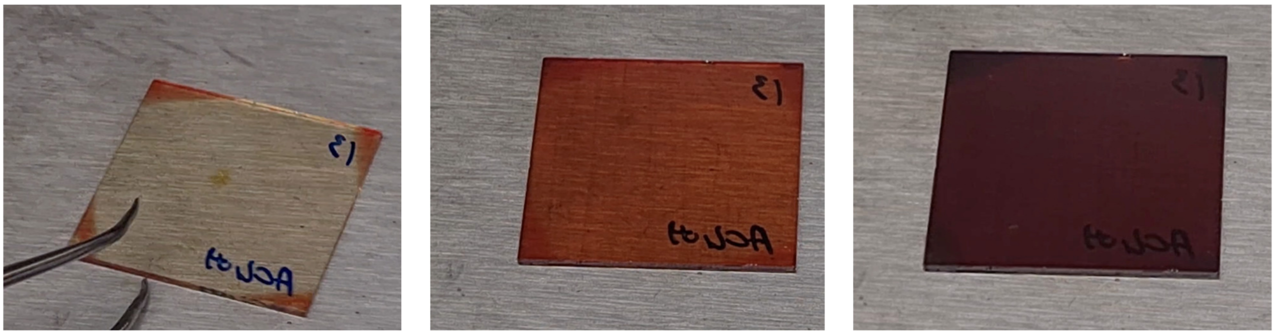

Wet film First stage: red film (~5 s) Second stage: dark film (~60 s)

Figure S5. Different stages of the crystallization of $(C12)_2MA_3Pb_4I_{13}$. The wet film first turns red (wide bandgap) then a darker color.

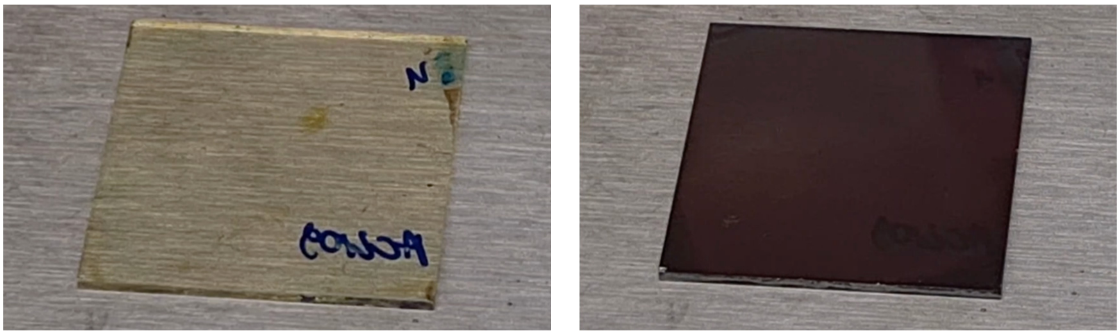

Wet film | First stage: dark film (~15 s)

Figure S6. Crystallization of $(C4)_2MA_3Pb_4I_{13}$. The film directly turns dark.
